# Could ChatGPT and co. replace forensic experts? A comparative study on medical liability expertise

**DOI:** 10.1007/s00414-026-03777-2

**Published:** 2026-03-26

**Authors:** Antoine Bérar, Jean-Sébastien Allain, Renaud Bouvet

**Affiliations:** 1 Department of Legal & Forensic Medicine, Rennes University Hospital, Rennes, F-35000 France; 2 Department of Polyvalent Medicine, South Brittany Hospital, Lorient, F-56100 France; 3 UMR CNRS 6262 Institut de l’Ouest : Droit et Europe (IODE), University of Rennes, Rennes, F-35000 France

**Keywords:** Expert testimony, Forensic sciences, Generative artificial intelligence, Liability, Malpractice

## Abstract

Artificial intelligence is expected to play an increasingly significant role in both medicine and law. The performance of generative artificial intelligence (GAI) models observed since 2022 may encourage consideration of their use in forensic expertise. We evaluated the ability of such models, in 2025, to conduct medical liability expertises. Nine fictional clinical cases describing the medical management of patients treated by a healthcare professional or institution were submitted to three GAI models (ChatGPT-4 Turbo, Gemini, and Mistral AI). The models were prompted to determine whether or not medical malpractice had occurred. Each model was queried five times for each case, and the responses were compared to the conclusions of a panel of three experts. Out of 135 requests, the conclusions of the GAI models aligned with those of the expert panel in 86 instances. The discrepancies were partly due to false negatives, where shortcomings were missed by the models, and to false positives, where deficiencies were identified that experts did not acknowledge. In other cases, they reflected differences similar to those that might arise between human experts. All models provided contradictory answers for the same case at least once. Taken together, these risks of false negatives, false positives and internal inconsistencies presently prevent the use of GAI models in conducting forensic medical assessments. Beyond these concerns, we doubt that the current functioning of GAI models, which relies on probabilistic content generation, is suited to forensic expertise and its demand for rigorous reasoning. While GAI tools may eventually complement human expertise, the ultimate assessment and conclusions should remain the prerogative of trained forensic medical experts.

**Supplementary information:**

The online version contains supplementary material available at 10.1007/s00414-026-03777-2.

## Introduction

Forensic medicine has long played a central role in judicial decision-making. As early as the 16th century, the French surgeon Ambroise Paré defined it as “the art of providing sworn reports in court”, a definition that still captures the core mission of forensic experts today: assisting judicial authorities by addressing technical medical questions that fall outside their field of expertise. Among the various domains of forensic medicine, medical liability expertise occupies a particular position, as it often relies primarily on the analysis of written medical records rather than on direct clinical examination.

In parallel, the rapid development of generative artificial intelligence (GAI) in recent years has raised questions about their potential applications in medicine and law. Large language models are specifically designed to process and generate text, making them theoretically well suited to tasks involving document analysis and synthesis of complex information. As a result, their possible use in forensic contexts—particularly for expert assessments based on documentary evidence—has begun to attract increasing attention. Recent studies have explored the application of artificial intelligence (AI) in forensic and legal medicine. A seminal contribution highlighted the potential and limitations of the integration of AI into healthcare, emphasizing both the performance gains and the ethical and legal concerns raised by such use cases [1]. Other works have examined the use of AI in forensic sciences more broadly, including case analysis, with encouraging but heterogeneous results [2, 3]. Together, these studies suggest that while GAI models may offer valuable support in forensic contexts, their reliability and consistency remain open questions.

Medical liability expertise represents a particularly demanding use case for GAI models. It requires not only medical knowledge, but also the ability to assess professional conduct in light of established scientific standards, clinical context, and accepted practices at the time of care. The expert’s task is not merely to identify diagnostic or therapeutic errors, but to determine whether the care provided complied with established medical knowledge and, if not, whether a fault can reasonably be attributed to the healthcare professional or institution. Despite growing interest in AI-assisted forensic expertise, empirical data evaluating the performance of GAI models in this specific domain remain scarce.

The primary objective of this study was to assess the current ability of several GAI models to conduct forensic medical liability evaluations similar to those entrusted to medical experts. The secondary objective was to analyze the variability of the models’ responses to the same query, in order to explore their consistency and reliability in a forensic context.

## Methods

Nine fictional clinical cases, each one to two pages long, were drafted by one of the authors (AB) and are available as supplemental material. These cases describe the medical management of patients treated by a healthcare institution or professional, and cover various medical scenarios: amoxicillin allergy (case 1), giant cell arteritis (case 2), colorectal cancer (case 3), COVID-19 (case 4), pulmonary embolism (case 5), subarachnoid hemorrhage (case 6), herpetic meningoencephalitis (case 7), clozapine-induced agranulocytosis (case 8), and cauda equina syndrome of discogenic origin (case 9). The liability of the institution or professional is supposed to be called into question, without specifying the alleged fault. A panel of three experts (AB, RB, JSA), all qualified in the field of personal injury and forensic medical expertise, analyzed the cases. AB is a forensic physician and internist, RB a full professor of forensic medicine and health law, and JSA an internist. None specializes in AI. The panel determined whether medical malpractice had occurred and, if so, its nature. Decisions were made following collective deliberation, before querying the GAI models. Consensus was reached in eight of the nine cases. In case 6 (subarachnoid hemorrhage), uncertainty led the panel to consult a full professor specializing in emergency medicine. The cases were subsequently submitted to three GAI models: ChatGPT-4 Turbo (developed by OpenAI), Gemini (developed by Google DeepMind), and Mistral AI [4–6]. Each prompt contained a single instruction, reproduced identically for all queries, followed by the fictional clinical case. The instruction was formulated as follows:

*“I want you to indicate whether*,* in the case below*,* the medical care provided was in accordance with established medical knowledge. You may describe your analysis of the case*,* but at the end*,* you must provide a clear answer.”*

For ambiguous responses, the following clarification was requested: “*Be more specific. Was there a fault or not?*”

We submitted each case five times to each model. All five queries for the nine cases were conducted on February 6, 2025 for each of the three models.

This study did not require any regulatory procedures, as no personal data was processed. The fictional patients were identified as Mr. or Mrs. X, while healthcare professionals were referred to as A, B, C… in order of appearance. Hospitals were not named.

## Results

The results are presented in Table 1. Out of 135 requests, the conclusions of the GAI models were of the same nature as those of the panel of experts in 86 instances.

**Table 1:** Responses from ChatGPT, Gemini, and Mistral AI in nine fictional cases dealing with medical liability, compared to the conclusions of a panel of experts

	Expert Panel	ChatGPT (*n* = 5)	Gemini (*n* = 5)	Mistral AI (*n* = 5)
Case 1 – Amoxicillin Allergy	Fault (treatment mistake)	Fault (treatment mistake, *n* = 5)	No fault (*n* = 5)	Fault (treatment mistake, *n* = 4)
				No fault (*n* = 1)
Case 2 – Giant Cell Arteritis	No fault	No fault (*n* = 5)	No fault (*n* = 5)	No fault (*n* = 5)
Case 3 – Colorectal Cancer	No fault	No fault (*n* = 5)	No fault (*n* = 5)	No fault (*n* = 5)
Case 4 – COVID-19	Fault (treatment mistake)	No fault (*n* = 5)	No fault (*n* = 5)	No fault (*n* = 5)
Case 5 – Pulmonary Embolism	Fault (delayed diagnosis)	Fault (delayed diagnosis, *n* = 5)	Fault (delayed diagnosis, *n* = 5)	Fault (delayed diagnosis, *n* = 5)
Case 6 – Subarachnoid Hemorrhage	Fault (delayed diagnosis)	Fault (delayed diagnosis, *n* = 4)	Fault (delayed diagnosis, *n* = 5)	Fault (delayed diagnosis, *n* = 5)
		No fault (*n* = 1)		
Case 7 – Herpetic Meningoencephalitis	No fault	No fault (*n* = 3)Fault (delayed diagnosis, *n* = 2)	Fault (delayed diagnosis, *n* = 4; follow-up failure, *n* = 1)	Fault (delayed diagnosis, *n* = 5)
Case 8 – Clozapine-Induced Agranulocytosis	Fault (treatment mistake)	No fault (*n* = 5)	No fault (*n* = 3)	No fault (*n* = 4)
			Fault (follow-up failure, *n* = 2)	Fault (follow-up failure, *n* = 1)
Case 9 – Cauda Equina Syndrome	No fault	No fault (*n* = 5)	No fault (*n* = 5)	No fault (*n* = 5)

### Expert panel

The expert panel deemed four out of the nine cases to be in compliance with established medical knowledge (cases 2, 3, 7, and 9). The management described in case 1 was judged non-compliant due to an obvious treatment error: amoxicillin was prescribed to a patient with a known allergy to the drug (angioedema). In case 4, the failure of a general practitioner to prescribe nirmatrelvir/ritonavir (Paxlovid^®^) for a patient over 65 years old with COVID-19 was considered inconsistent with a guideline previously published by the *Haute Autorité de Santé* (French national authority for health) [7]. In case 5, the expert panel identified a failure in the etiological diagnosis of dyspnea: the symptom should have been explored further before being labeled as psychogenic, and its persistence—combined with deteriorating vital signs (tachycardia, low-grade fever, oxygen saturation approaching the lower limit of normal)—should have warranted a clinical reassessment rather than a telephone advice. In case 6, the absence of brain imaging (preferably a CT scan) in response to a sudden headache in a non-migraine patient was considered a shortcoming. Finally, in case 8, the monitoring of the white blood cells under clozapine followed the recommendations, but the prescription of this antipsychotic was considered too early. Indeed, clozapine was introduced as a second-line treatment, whereas the marketing authorization for this medication states that its prescription should only be considered in schizophrenic patients after the failure of two other antipsychotics, or in cases of severe neurological side effects that cannot be corrected.

### ChatGPT

ChatGPT’s conclusions aligned with those of the expert panel in cases 1, 2, 3, 5, and 9. In case 4 (COVID-19), ChatGPT did not discuss the indication for nirmatrelvir/ritonavir (Paxlovid^®^) and did not identify any fault in the different stages of management. In case 6 (subarachnoid hemorrhage), its conclusions matched those of the expert panel in four out of five queries. In the remaining query, it concluded that no fault had occurred despite an ambiguous response: “*The initial management of the patient was in accordance with established medical knowledge*,* but there was a delay in performing a brain CT scan. The correct diagnosis of SAH* [subarachnoid hemorrhage] *was made during the patient’s second emergency visit*,* where the management was appropriate and rapid. In summary*,* there was no medical fault in the management*,* although headaches should always be approached with caution*,* especially in cases of recurrence*,* and a CT scan could have been considered earlier.*” In case 7 (herpetic meningoencephalitis), ChatGPT identified a diagnostic delay in two queries (e.g., “*The management of Mr. X was not entirely in accordance with established medical knowledge initially*,* due to a delay in identifying the viral cause of his symptoms. The initial management did not sufficiently consider the possibility of herpetic encephalitis despite alarming symptoms. However*,* once the lumbar puncture was performed and the diagnosis was made*,* treatment and follow-up were appropriate and in line with current recommendations for viral encephalitis.*”). On the other hand, in the other three queries, it judged the management to be compliant (e.g., “*The initial management of Mr. X was in accordance with established medical knowledge*,* considering the absence of severe signs of brain pathology and the diagnosis made at the first consultation. However*,* the rapid reassessment at the second consultation allowed for the correct diagnosis of herpetic encephalitis*,* and therapeutic management was appropriate. While the delay before definitive management may have slightly prolonged disease progression*,* the therapeutic response appears to be in line with current recommendations. Final response: The management was in accordance with established medical knowledge*,* with appropriate initial handling and relevant reassessment leading to appropriate treatment.*”). In case 8 (clozapine-induced agranulocytosis), ChatGPT did not identify any fault in the prescription of clozapine or at any other stage of the patient’s management. Across all 45 queries, 5 requests for clarification (“*Be more specific*”) were necessary.

### Gemini

**Fig. 1:**
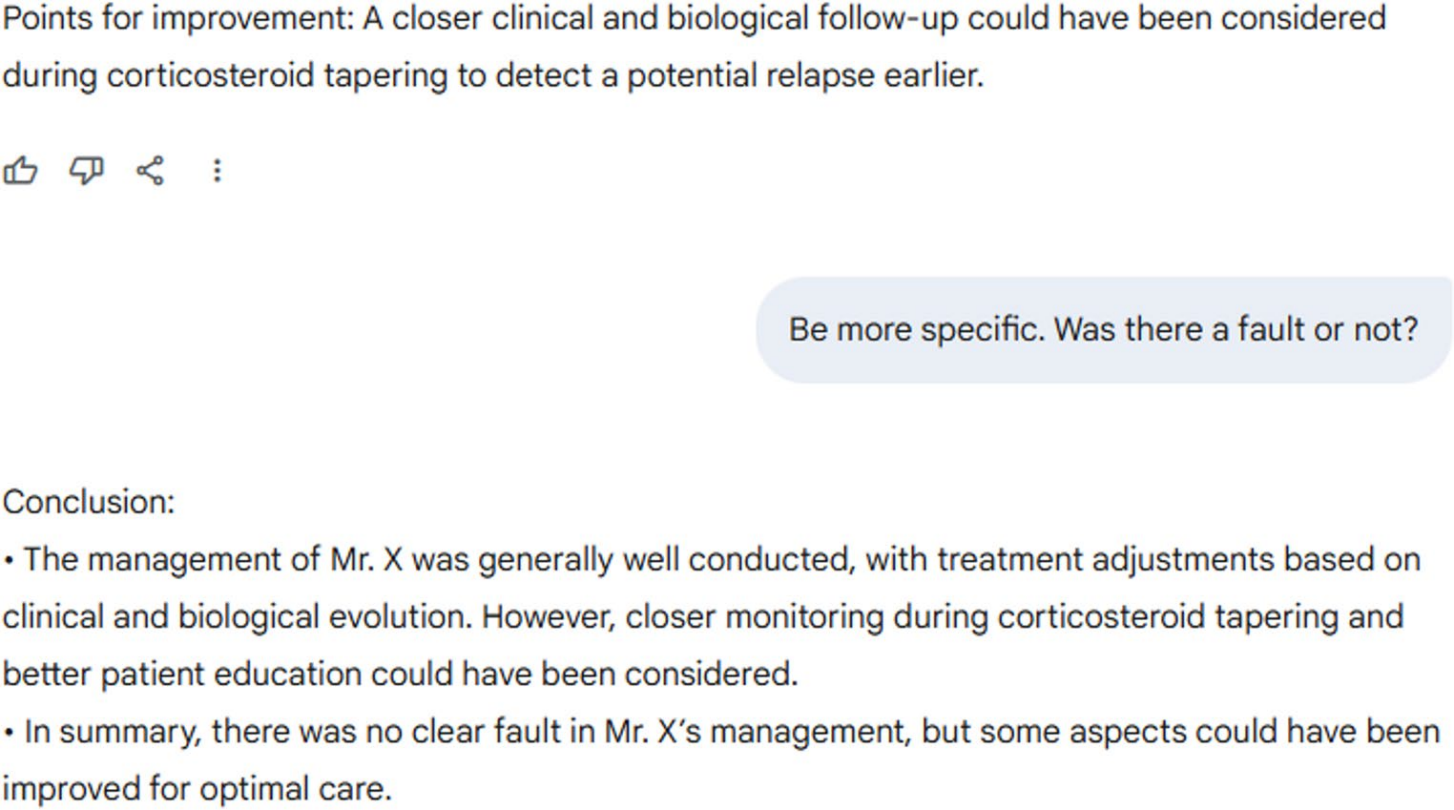
“Points for improvement” suggested by Gemini in case 2 (giant cell arteritis).(The figure displays a translation in English of the chat with Gemini, originally in French.)

Gemini’s conclusions matched those of the expert panel in cases 2, 3, 5, 6, and 9. In case 1 (amoxicillin allergy), Gemini did not mention the patient’s allergy history and did not consider it a contraindication to amoxicillin in any of the five queries. In case 4 (COVID-19), it did not discuss the indication for nirmatrelvir/ritonavir (Paxlovid^®^) and did not identify any fault in the management. In case 7 (herpetic meningoencephalitis), Gemini consistently identified a fault in all queries, citing a diagnostic delay due to a failure in the patient’s initial emergency department visit (four queries) or inadequate follow-up after discharge (one query). In case 8 (clozapine-induced agranulocytosis), Gemini did not identify any fault in three queries. In the other two, it pointed to a failure in monitoring the schizophrenic patient, who was lost to follow-up after missing an outpatient appointment. Across all 45 queries, 9 requests for clarification were necessary. Gemini stood out for its tendency to suggest “points for improvement”, as illustrated in case 2 (see Fig. 1).

### Mistral AI

Mistral AI’s conclusions aligned with those of the expert panel in cases 2, 3, 5, 6, and 9. It judged the management of case 1 (amoxicillin allergy) as compliant in one query but identified a fault in the prescription of amoxicillin in the other four. In case 4 (COVID-19), it did not discuss the indication for nirmatrelvir/ritonavir (Paxlovid^®^) and did not identify any fault in the management. In case 7 (herpetic meningoencephalitis), it consistently pointed to a diagnostic delay. In case 8 (clozapine-induced agranulocytosis), Mistral AI did not identify any fault in four queries. In one query, however, it pointed to inadequate follow-up, as the schizophrenic patient was lost to follow-up after missing an outpatient appointment. Across all 45 queries submitted to Mistral AI, four requests for clarification were necessary.

## Discussion

GAI is increasingly explored in medicine, with studies demonstrating strong performance on knowledge retrieval and problem-solving tasks. Large language models such as ChatGPT and Med-PaLM 2 have achieved high scores on medical licensing examinations and other structured tasks that rely on well-defined questions and clear correct answers [8, 9]. These findings highlight the potential of GAI to support clinical reasoning and knowledge synthesis. However, most prior work has focused on scenarios with explicit solutions, while real-world medical decision-making often requires interpretation, judgment under uncertainty, and consideration of context-specific standards. In particular, the application of GAI to forensic medical expertise, which involves assessing patient management against established standards, identifying potential malpractice, and exercising nuanced judgment, has not been systematically studied.

To address this gap, we presented nine fictitious clinical cases to three GAI models, providing them with a simple instruction dealing with medical liability: they were asked to determine whether a fault existed in the medical management of a patient. ChatGPT and Gemini were chosen primarily due to their widespread reputation, while Mistral AI was selected for being an European-based model and for its supposedly lower environmental impact compared to these competitors [10]. GAI naturally produces different outputs for the same input. Hence, the single prompt was tested multiple times to study the variability of responses generated by each model. We conducted all five queries on the same date to minimize the impact of rapid evolution of training data. Our findings lead to several observations, primarily regarding the comparison of the expert panel’s responses with those of the language models, and secondly, the variability of responses within each model.

### Comparison of responses

In four of the nine cases examined (cases 2, 3, 5, and 9), the conclusions drawn by the GAI models were entirely consistent with those of the expert panel. These four cases share a fairly unambiguous nature, as the medical management was clearly either non-compliant (case 5) or compliant (cases 2, 3, and 9) with established scientific knowledge. The ability of GAI models to detect major medical shortcomings may seem promising based on these results. However, the discrepancies observed in case 1 temper these expectations: Gemini failed to account for the patient’s amoxicillin allergy (in all five requests), as did Mistral AI (in one request), despite this being a clear contraindication to prescribing the drug. These false negatives (due to an inability to recognize a deficiency) represent, in our view, the most aberrant responses in this study. Similarly, case 8 also highlights the inability of GAI models to detect certain shortcomings. It is widely accepted that clozapine is not indicated in the early stages of schizophrenia treatment, but is reserved for treatment-resistant schizophrenia [11]. Even though the definition of the latter is not entirely consensual, it generally relies on the failure of two different antipsychotic drugs as a minimum criterion [12]. It would be surprising if these well-established concepts were not part of the training data for GAI models. Yet, none of the three models addressed this issue. In the same case, Gemini (twice) and Mistral AI (once) pointed out a lack of follow-up, as the patient was lost after missing an appointment. This conclusion seems excessively severe and was not upheld by the panel of experts. In our view, these results constitute false positives, as they represent shortcomings identified by the models that do not correspond to actual faults in medical management. All these false negatives and false positives could have serious and ethically unacceptable consequences in the context of forensic evaluations intended to guide judicial decisions. Therefore, these results alone limit the trust that can be placed in GAI models for conducting, today, forensic medical assessments.

Cases 6 and 7 also present partial discrepancies between the conclusions of the expert panel and the GAI models: ChatGPT diverged in case 6, while all three models tested diverged in case 7. However, in these instances, the conclusions drawn by the GAI models reflect perspectives that could plausibly be adopted by real experts. Case 6 involved a headache that improved with level 1 analgesics and nonsteroidal anti-inflammatory drugs, later revealed to be due to a subarachnoid hemorrhage. This atypical evolution of the headache could mitigate the liability of the healthcare institution, even though the expert panel deemed that a brain scan was still warranted given the nature of the pain (sudden and holocranial) and the patient’s age [13, 14]. In case 7, the expert panel did not find any fault in the medical management. Specifically, it deemed the diagnosis of herpetic meningoencephalitis particularly challenging during the patient’s initial emergency department visit, given the available clinical data. Mistral AI consistently concluded that the medical management was inadequate due to the absence of further investigations at that stage. To further examine this issue, we submitted five additional requests to Mistral AI, but this time, the case description stopped at the patient’s discharge after their first emergency visit. Under these conditions, Mistral AI systematically concluded that no fault had occurred—whereas, when presented with the full case, it had deemed the management deficient. This suggests that information about the patient’s subsequent health evolution influenced its response. Nonetheless, the absence of thorough investigation during the first emergency visit was arguably questionable, and an expert might have similarly deemed the medical management as faulty. Some AI-generated responses could therefore be considered legitimate within a spectrum of possible expert opinions, provided they are sufficiently substantiated. The divergences observed between the expert panel and the GAI models may thus resemble the variations sometimes found among medical experts evaluating the same case. In fact, it cannot be ruled out that differences might have emerged within the panel of experts itself had the cases been assessed independently by each of its three members. The phenomenon of groupthink, driven by a desire for harmony and conformity within a group, tends to smooth over differences of opinion and suppress disagreement [15, 16]. The apparent uniformity of the panel’s conclusions may therefore conceal more substantial nuances in judgment than it appears to reflect.

In case 4, the panel of experts identified a fault based on a specific text: it regretted the absence of a prescription for nirmatrelvir/ritonavir (Paxlovid^®^) in a 77-year-old polymorbid patient with COVID-19, despite a recommendation from the French national authority for health advocating its use in patients of that age. This finding can be interpreted in several ways. First, it is unknown whether this recommendation, or other texts imitating or reiterating it, was included in the training data of language models. This highlights the crucial role of training data in shaping the generated results. Second, assuming this text is part of the training data, this result could indicate that GAI models may fail to detect faults when patient management only contradicts a limited number of recommendations. They may struggle to identify these texts within their vast training data and/or to assign them greater weight than data not originating from scientific societies or health authorities. Another possible explanation relates to the geographic variability of training data. While the query was formulated in French and from the French territory, it did not explicitly specify that the clinical case took place in France. Yet, the French recommendation on nirmatrelvir/ritonavir is unlikely to be universally shared. In countries with more limited access to healthcare, such guidelines for the drug’s use may not be in place. In this light, the responses provided by the three GAI models are likely to be accurate depending on the geographic perspective. Finally, the fact that the recommendation considered by the panel of experts dates from December 2023 raises questions about how GAI models incorporate the most recent data. Nirmatrelvir/ritonavir was authorized in the United States on December 22, 2021, followed by a European authorization a few weeks later [17, 18]. The recommendation on nirmatrelvir/ritonavir from the French National Authority for Health is, by nature, contrary to those predating the market introduction of this drug. It remains unclear whether language models prioritize recent information over older, conflicting data. Moreover, we do not know how they understand the concept of “scientifically established data”, which is a topic of debate within the legal community itself, particularly regarding the meaning of the term “established” [19–21]. This also illustrates a structural limitation of GAI models: they do not inherently operate within a clearly defined legal or regulatory framework, but instead generate responses based on a probabilistic synthesis of heterogeneous sources. This makes them sensitive to ambiguities in geographic or temporal context and potentially incompatible with the localized and time-dependent nature of medical liability assessment [22]. While prompting strategies can explicitly instruct a model to adopt a specific legal or regulatory framework, such constraints remain external, fragile, and dependent on correct interpretation and faithful adherence by the model, rather than being intrinsically embedded in its reasoning process.

Hence, this issue is not limited to the example of Paxlovid^®^. In many areas of medicine, the assessment of a potential fault critically depends on both the geographical and temporal context of the applicable standards of care. During the COVID-19 pandemic, for instance, recommendations regarding treatments such as hydroxychloroquine, azithromycin, remdesivir, corticosteroids, or anticoagulation evolved rapidly and sometimes differed between countries and health authorities [23, 24]. Similarly, in the diagnostic workup of subarachnoid hemorrhage, the respective roles of early CT scan, lumbar puncture, and CT angiography have changed over time and remain subject to international variations. In all these situations, the determination of medical liability requires a precise reconstruction of what constituted the accepted standard of care in a specific place and at a specific time. This localized and time-dependent dimension might be difficult to capture for GAI models.

### Variability of language model responses

Among the 27 groups of requests, five exhibited variability in the conclusions generated by the GAI models (two for ChatGPT, one for Gemini, and two for Mistral AI). While different expert opinions may be justifiable for the same case, the fact that a single GAI model generates conflicting conclusions is perplexing. Rather than reflecting distinct expert perspectives, this phenomenon resembles a single judicial expert providing inconsistent conclusions when questioned multiple times on the same case. This variability partly reflects the very functioning of GAI, whose probabilistic nature inherently produces non-reproducible results. Yet, the fact that variability was predominantly observed in the most complex cases (four out of five variable responses concerned cases 6, 7, and 8) suggests that greater consistency might be achieved with improved GAI performance. Regardless, this result further limits the feasibility of using GAI as a forensic medical assessment tool at present. A judicial decision cannot depend on a coin tossed in the air, which may land on either side.

### Limitations and methodological considerations

Our study has several limitations. Firstly, the clinical cases submitted to GAI models were fictional cases. Although they were designed to resemble real-world situations, they are likely less complex than actual forensic evaluations and may therefore be easier to assess. Additionally, these cases were synthetic and generally focused on a specific medico-legal issue, whereas real medical records typically require synthesizing scattered data, containing a large volume of information that could distract GAI models from the core medico-legal issue. GAI models might struggle to synthesize extensive records effectively, focus on insignificant details, or overlook important elements (such as the amoxicillin allergy mentioned in Case 1), leading to inappropriate conclusions. Moreover, the fictional cases reflect the specialties with which the author was most familiar. They do not cover the entire spectrum of medicine, thereby limiting the generalizability of the study’s results. A single prompt formulation was used throughout the study, but the relevance of GAI models’ responses may vary depending on how the prompt is phrased. Likewise, personalization features available in some models, such as ChatGPT, were not utilized, although incorporating personalized information about the user and their expectations might improve the quality of generated responses.

Additional methodological limitations relate to the scope and design of the evaluation. Only the variability of GAI model responses was assessed, whereas the variability of expert opinions was not formally examined. As discussed earlier, expert perspectives may differ on complex issues like medical liability assessment. Lastly, the instructions given to GAI models focused solely on the presence or absence of malpractice, without requiring an analysis of the causal link between malpractice and harm or an evaluation of the damage—yet these are crucial aspects of forensic expertise intended to inform judicial authorities.

### Ethical and legal implications

Ultimately, it is premature to predict the end of physicians’ monopoly on forensic expertise tasks. GAI models exhibit a risk of false negatives and false positives, as well as substantial variability in their responses, making their use by judicial authorities inconceivable in the near future. Even if the current limitations of GAI models were overcome, potentially insurmountable barriers would remain in equating a GAI-generated conclusion with that of a human expert. It is already known that ChatGPT and Med-PaLM 2 perform well on exams similar to those taken by medical students [8, 9]. However, when analyzing a situation and assessing the actions of a fellow practitioner, a medical expert does not rely solely on academic knowledge: they also draw upon their professional experience and the lessons learned from it—in other words, they use *experiential knowledge* [25]. While not limited to it, experiential knowledge is rooted in *experience*, which GAI models inherently lack. It is through this experience and the knowledge derived from it that an expert can analyze a given situation in all its uniqueness, sometimes justifying deviations from recommendations that might seem applicable to the case. Furthermore, the expert is able to put themselves in the shoes of a “reasonably diligent physician” without requiring an illusory mastery of all existing medical knowledge. While GAI models cannot accumulate personal experience, they can be trained on data providing access to *discourse* about experience, such as professional discussion forums or clinical cases. Perhaps this massive and continuously generated training data could compensate for AI’s lack of real-world experience, or even, in an extreme perspective, confer them a significant competitive advantage over human experts, whose experience is constrained by the temporal and spatial limits of human life. Moreover, human experts themselves may sometimes wrongly extrapolate their own experience beyond its domain of validity —whereas ideally, such knowledge should not be generalized to different contexts.

Beyond the issue of experience, a key limitation of current GAI models in the context of forensic expertise is their opacity, often referred to as the “black box” problem [26, 27]. These models generate probabilistic outputs based on patterns learned from massive datasets, yet the internal reasoning steps remain largely inaccessible to users and even to developers in many cases. This lack of transparency poses challenges in high-stakes domains such as medical liability, where decisions must be justifiable, contestable, and traceable. Research in explainable artificial intelligence (XAI) has sought to mitigate these concerns by providing methods to visualize or approximate the functioning behind model outputs, but they do not fully resolve the inherent uncertainty and probabilistic nature of language model reasoning [28, 29]. Consequently, relying on GAI outputs for forensic judgments without human oversight remains ethically and legally problematic.

Finally, the use of GAI in forensic expertise raises unresolved issues of responsibility and accountability. When an expert opinion is influenced by an algorithmic output, it becomes unclear who should bear responsibility in case of error: the human expert, the institution that provided or mandated the use of the tool, or the developer of the model [30]. Moreover, the risk of automation bias may lead human experts to over-trust GAI outputs, especially when they are formulated in fluent and authoritative language [31]. In the context of forensic expertise, this could lead experts to give undue weight to GAI-generated analyses, even when they are incorrect, incomplete, or poorly adapted to the specific legal and clinical context. Unlike human experts, language models do not possess professional responsibility, are not bound by ethical codes, and cannot be cross-examined. As recently emphasized in the legal and ethical literature, the introduction of GAI into judicial or quasi-judicial contexts therefore requires extreme caution, and must in any case preserve the central role of the human expert in both the reasoning process and the final conclusions [32, 33]. Taken together, these considerations suggest that, at least in their current and foreseeable forms, GAI models should not be viewed as substitutes for forensic medical experts.

### Conclusion and future perspectives

The limitations identified in this study do not preclude all future uses of GAI in the forensic field. Rather than replacing human experts, a more realistic and ethically acceptable perspective may lie in the development of hybrid models, in which GAI tools support—but do not substitute for—expert reasoning. Such tools could, for example, assist in structuring large and complex medical records, identifying relevant guidelines, or highlighting potential points of discussion, while leaving the final assessment, interpretation, and conclusions to trained forensic medical experts.

Future research should therefore focus on clearly defined use cases, the construction of domain-specific and geographically contextualized training datasets, and the integration of explainability and traceability mechanisms that allow users to understand how conclusions are generated. Establishing rigorous evaluation frameworks and governance rules will also be essential if GAI tools are to be safely incorporated into forensic practice. Under these conditions, GAI may eventually contribute meaningfully to forensic expertise, not as a shortcut to expert judgment, but as a carefully supervised instrument serving it.

## Supplementary information

Below is the link to the electronic supplementary material.

Supplementary File 1 (PDF 119 KB) The nine clinical cases, in French.

Supplementary File 2 (PDF 219 KB)

Supplementary File 3 (PDF 209 KB)

Supplementary File 4 (PDF 117 KB)

Supplementary File 5 PDF 216 KB)

Supplementary File 6 (PDF 205 KB)

Supplementary File 7(PDF 209 KB)

Supplementary File 8 (PDF 226 KB)

Supplementary File 9 (PDF 199 KB)
