# Supplementary material for: Could ChatGPT and co. replace forensic experts? A comparative study on medical liability expertise
Source: Int J Legal Med. 2026 Mar 26;140(4):2533–41. doi: 10.1007/s00414-026-03777-2 (PMC13275606; doi:10.1007/s00414-026-03777-2)
Supplement: Supplementary file 1 — (PDF 119 KB) The nine clinical cases, in French. [file 414_2026_3777_MOESM1_ESM.pdf]

A partir du 18 décembre 2022, Monsieur X., 57 ans, présente des brûlures mictionnelles associées à une pollakiurie et à un syndrome polyuro-polydipsique. Il a pour antécédents une hypertension artérielle, un tabagisme sevré, des consommations occasionnelles d'alcool et une chirurgie du genou gauche. Il a des allergies connues à l'amoxicilline (œdème de Quincke) et à certains AINS (sans précision).

Il consulte son médecin traitant le 20 décembre 2022. Un traitement par COTRIMOXAZOLE est prescrit mais non pris. Le même jour, un ECBU est réalisé. Le 21 décembre, des douleurs lombaires bilatérales apparaissent, ainsi que de la fièvre, des sueurs et des frissons. Le bilan biologique du 23 décembre montre un syndrome inflammatoire (CRP 292 mg/l, PNN 16 G/l). Monsieur X. est adressé aux urgences du CHU. A l'admission aux urgences, la tension artérielle est à 157/97 mmHg, la fréquence cardiaque à 101/min, la température à 39,4°C, la saturation en oxygène à 97 % en air ambiant. Il n'y a pas d'instabilité hémodynamique. Le bilan biologique montre une élévation de la créatininémie (148 µmol/l). Des hémocultures et un ECBU sont prélevés. L'uroscanner révèle une pyélonéphrite bilatérale (foyers de néphrite bilatéraux), sans complication. Sur le plan thérapeutique, une antibiothérapie probabiliste par CEFOTAXIME est débutée (J1 = 24/12/2021). Une réhydratation intraveineuse est entreprise (NaCl 0,9 % 2 litres par 24 heures).

Monsieur X. est transféré dans l'unité de médecine polyvalente le 25 décembre pour la suite des soins. L'ECBU se révèle positif à *Escherichia coli* multi sensible. L'antibiothérapie par CEFOTAXIME est relayée par AMOXICILLINE le 27 décembre. Le 27 décembre au soir, Monsieur X. présente une réaction anaphylactique de grade 2 après l'administration orale d'AMOXICILLINE. Le 27 décembre 2022 à 19 heures 49, Monsieur A., interne, note :

> « *Réaction anaphylactique de grade 2 après l'administration d'amoxicilline.*
> *Rash cutané (apparu vers 18h45), hypotension artérielle avec PAM 52 mmHg (à 19h). SpO2 94% en air ambiant. Pas de dyspnée, pas de dysphonie. Auscultation pulmonaire claire.*
> *Administration adrénaline (0,1 mg IV) à 19h03. Restauration rapide et stabilisation de la PAM (83 mmH à 20h30). Traitement par Ceterizine pour le prurit.*
> *Bilan biologique d'anaphylaxie fait (premier prélèvement, histamine + tryptase, vers 19h30).*
> *Arrêt amoxicilline.*
>
> *Vu par l'équipe de réanimation. Poursuite de la surveillance dans le service.* »

Le 28 décembre 2022 à 10 heures 16, le même interne note :

> « *Va bien ce matin. Quelques lésions cutanées persistantes, non prurigineuses. TA normale, pas de signe d'hypoperfusion périphérique.*
> *Pas de signe fonctionnel urinaire ni douleur lombaire.*
> *Relais par BACTRIM FORTE (pas d'allergie connue au COTRIMOXAZOLE).* »

L'évolution est favorable par la suite. Les signes fonctionnels urinaires et la fièvre disparaissent. La fonction rénale se normalise sous hydratation intraveineuse : l'urée est à 4,6 mmol/l et la créatininémie à 100 µmol/l le 28 décembre. L'insulinothérapie IVSE est arrêtée le 29 décembre et une réévaluation du traitement du diabète est entreprise.

Monsieur X. quitte l'hôpital le 30 décembre 2022, avec une prescription de BACTRIM à poursuivre jusqu'au 7 janvier inclus.
