# Supplementary material for: Could ChatGPT and co. replace forensic experts? A comparative study on medical liability expertise
Source: Int J Legal Med. 2026 Mar 26;140(4):2533–41. doi: 10.1007/s00414-026-03777-2 (PMC13275606; doi:10.1007/s00414-026-03777-2)
Supplement: Supplementary file 2 — (PDF 219 KB) [file 414_2026_3777_MOESM2_ESM.pdf]

Monsieur X. est âgé de 61 ans. Il a pour antécédents une colique néphrétique droite, d'origine lithiasique, et un rétrécissement aortique.

A partir du début de l'année 2022, Monsieur X. présente une altération de l'état général : asthénie avec fatigabilité, amaigrissement (9 à 10 kg sur trois mois), anorexie. Il décrit des sueurs nocturnes et une toux sèche évoluant depuis plusieurs mois. Le bilan réalisé par son médecin traitant met en évidence un syndrome inflammatoire biologique. Le scanner thoraco-abdomino-pelvien du 30 mars ne montre pas de foyer infectieux ni de syndrome tumoral.

Il est adressé aux urgences du CHU le 11 avril devant la persistance du tableau clinique. Il est apyrétique (37,4°C). Il rapporte une symptomatologie douloureuse migratrice, située au niveau du bras gauche au moment du passage aux urgences. L'examen clinique est normal en dehors du souffle cardiaque systolique de rétrécissement aortique. Il est hospitalisé en médecine interne. L'examen d'entrée est renseigné par l'interne :

> « *TA 112/72 mmHg, FC 105/min, T 37,3°C, SpO2 99 % en air ambiant.*
> *Pas de signe d'hypoperfusion périphérique.*
> *Examen cardiovasculaire : pas de douleur thoracique, pas de dyspnée de repos. Pouls distaux perçus. Pas d'œdème des membres inférieurs, pas de turgescence jugulaire. Bruits du cœur réguliers, souffle systolique évocateur de rétrécissement aortique à tous les foyers, avec abolition du B2. Pression artérielle systolique au bras gauche inférieur de 40 mmHg à la pression artérielle diastolique du bras droit.*
> *Examen respiratoire : eupnéique en air ambiant. Toux sèche peu fréquente, lors de la parole. Auscultation pulmonaire claire et symétrique.*
> *Examen abdominal : pas de trouble du transit ni de signe fonctionnel urinaire (excepté une dysurie habituelle). Abdomen souple, dépressible et indolore.*
> *Examen neurologique : patient conscient, cohérent. Toux possible et flexion du chef possible. Pas de syndrome déficitaire. Réflexes ostéotendineux présents sauf en achilléen. Réflexes cutanés plantaires en flexion. Pas de signe de Babinski ni de signe de Hoffman.*
> *Examen ORL : à l'inspection buccale, pas d'anomalie de la luette, pas d'érythème pharyngé.*
> *Examen cutané : livedo aux deux cuisses. Télangiectasies au niveau du tronc, à hauteur de la poitrine et du flanc gauche.*
> *Artères temporales battantes, non indurées. Pas de céphalées. Pas d'hyperesthésie du cuir chevelu. Pas de claudication de la mâchoire.*
> *Aires ganglionnaires libres.*
> *Pas de signe périphérique d'endocardite.* »

Le bilan biologique confirme une élévation de la CRP (95,9 mg/l). Le bilan infectieux (hémocultures, ECBU, sérologies VIH, VHB, VHC, sérologie syphilis) est négatif. Le bilan immunologique ne révèle pas d'anomalie notable.

Le scanner thoraco-abdomino-pelvien ne met pas en évidence de foyer infectieux ni de syndrome tumoral. Il existe une aortite : épaississement pariétal circonférentiel étendu de l'aorte thoraco-abdominale, mesuré jusqu'à 3 mm d'épaisseur, sans anomalie de calibre associée.

Le diagnostic d'artérite à cellules géantes (maladie de Horton) est évoqué. Le TEP-scanner montre « *un hypermétabolisme modéré mais significatif des artères vertébrales, des artères sous-clavières, des artères fémorales et des parois de l'aorte thoracique et a minima abdominale* ». Ces résultats sont jugés en faveur d'une maladie de Horton. La biopsie d'artère temporale montre quant à elle des « *lésions de vascularite pan pariétale, granulomateuse, épithélio-gigantocellulaire, compatible avec une maladie de Horton.* »

Une corticothérapie est débutée. Monsieur X. quitte le CHU le 16 avril, avec la prescription de sortie suivante :

« *PREDNISONE :*
- *55 milligrammes le matin pendant 1 mois*
- *puis 45 milligrammes le matin pendant 2 semaines ;*
- *puis 35 milligrammes le matin pendant 2 semaines ;*
- *puis 30 milligrammes le matin pendant 1 semaine ;*
- *puis 25 milligrammes le matin pendant 1 semaine ;*
- *puis 20 milligrammes le matin pendant 1 semaine ;*
- *puis 15 milligrammes le matin et réévaluation* »

Monsieur X. est revu en consultation de médecine interne le 14 juin 2022 par le Docteur A. L'évolution est favorable : disparition de l'altération de l'état général, reprise de poids, disparition de la toux. La CRP est à 30 mg/l. Un dépistage et une prise en charge des complications de la corticothérapie est réalisée : dépistage et traitement d'un diabète cortico-induit ; vaccination contre le pneumocoque, la grippe et le Covid-19 ; dépistage de l'ostéoporose.

Une nouvelle consultation de médecine interne a lieu le 20 octobre 2022. Il n'est pas décelé de signe de rechute de la maladie de Horton. La CRP est à 4,8 mg/l. Le Docteur A. conclut : « *décroissance des corticoïdes progressive (ordonnance remise au patient) CORTANCYL 9 mg/jour, pendant 15 jours, puis décroissance prudente 9 mg les jours pairs, 8 mg les jours impairs pendant 15 jours, puis 8 mg pendant un mois, puis 7 mg les jours pairs, 8 mg les jours impairs, pendant 15 jours puis 7 mg pendant un mois. Ne pas arrêter le traitement brutalement et palier à 5 mg.* »

En janvier 2023, Monsieur X. présente de nouveau une altération de l'état général. Une toux sèche réapparaît. La CRP se majore, à 81 mg/l. Il est reçu en consultation par le Docteur A. Une rechute de la maladie de Horton est évoquée, et un traitement à visée d'épargne cortisonique par Tocilizumab est discuté.
