# Supplementary material for: Could ChatGPT and co. replace forensic experts? A comparative study on medical liability expertise
Source: Int J Legal Med. 2026 Mar 26;140(4):2533–41. doi: 10.1007/s00414-026-03777-2 (PMC13275606; doi:10.1007/s00414-026-03777-2)
Supplement: Supplementary file 3 — (PDF 209 KB) [file 414_2026_3777_MOESM3_ESM.pdf]

Monsieur X., 55 ans, a pour antécédents un diabète de type 2 insulino-dépendant, une polytoxicomanie sevrée et substituée par Methadone, et une stéatose hépatique.

Il est hospitalisé le 14 juin 2024 au centre hospitalier pour le bilan de son diabète. Le bilan d'entrée révèle une anémie microcytaire (hémoglobinémie 8 g/dl, VGM 70 fL), ferriprive (ferritinémie à 8 µg/l). Il n'y a pas d'atteinte des autres lignées (plaquettes 153 G/l, leucocytes 4,1 G/l). Deux perfusions de Ferinject sont administrées. La fibroscopie œsogastroduodénale réalisée le 24 juin 2024 par le Docteur A. montre :

> « *Aspect de gastrite atrophique. Pas de biopsies au vu de la tolérance médiocre.*
> *Indication à la réalisation sous AG d'une FOGD avec biopsies et d'une coloscopie.*
> *Il signale des rectorragies depuis quelques mois, pas de trouble du transit, pas de douleur abdominale, pas d'antécédent familial de cancer colorectal. N'a jamais eu de coloscopie.*
> *Explications données au patient sur les modalités, risques de l'examen.* »

Une coloscopie totale est réalisée le 12 juillet 2024 par le Docteur B., gastro-entérologue libéral. Elle révèle :

> « *Sténose circonférentielle de 4 à 5 cm de hauteur au niveau de la charnière recto-sigmoïdienne, s'étageant entre 18 et 24 cm de la marge anale environ, franchissable en frottement au coloscope adulte.*
> *On réalise de nombreuses biopsies sur la sténose d'allure tumorale.* »

Le scanner thoraco-abdomino-pelvien du 22 juillet 2024 montre un « *épaississement pariétal tissulaire circonférentiel irrégulier sub-sténosant au niveau de la charnière rectosigmoïdienne avec infiltration de la graisse adjacente et présence de multiples ganglions satellites* » et « *plusieurs nodules pulmonaires tissulaires, suspects de localisation secondaire.* »

Le TEP-scanner du 24 juillet 2024 montre :

> « *Présence d'une volumineuse masse hypermétabolique située au niveau de la charnière recto sigmoïdienne associée à la présence d'adénopathies hypermétaboliques lomboaortiques bilatérales et inter-aortico-caves. Présence de deux condensations parenchymateuses pseudonodulaires hypermétaboliques l'une située au niveau postérobasal droit la seconde au niveau du segment supérieur du lobe inférieur gauche en juxtascissural. Présence de deux foyers hypermétaboliques probablement ganglionnaires situés en sus-claviculaire gauche.* »

L'examen anatomopathologique des biopsies révèle un « *adénocarcinome lieberkühnien infiltrant moyennement différencié (grade 2/bas grade).* » Il n'y a pas d'argument immuno-histochimique en faveur d'une instabilité des microsatellites (p.MMR). Il existe une mutation du code 12 du gène KRAS ; il n'est pas décelé de mutation V600 du gène BRAF.

Monsieur X. est reçu en consultation d'annonce par le Docteur C. le 9 août 2024. Le compte-rendu de la consultation mentionne :

> « *Consultation d'annonce thérapeutique de chimiothérapie à visée de contrôle de la maladie sans possibilité de guérison, par une association de chimiothérapie par Folfox et thérapie ciblée par Avastin.*
> *Explication du protocole de chimiothérapie, des effets secondaires attendus, des médicaments pour pallier les effets secondaires notamment EMEND, de l'indication d'une pose de chambre*

implantable qui est prévue le 12 août : possibilité de début de traitement à partir du 19 août, avec début de l'Avastin à partir du 2e cycle.
Remise d'un plan personnalisé de soins. »
