# Supplementary material for: Could ChatGPT and co. replace forensic experts? A comparative study on medical liability expertise
Source: Int J Legal Med. 2026 Mar 26;140(4):2533–41. doi: 10.1007/s00414-026-03777-2 (PMC13275606; doi:10.1007/s00414-026-03777-2)
Supplement: Supplementary file 4 — (PDF 117 KB) [file 414_2026_3777_MOESM4_ESM.pdf]

Monsieur X., 77 ans, a pour antécédents un adénocarcinome du moyen rectum diagnostiqué en novembre 2022, ayant donné lieu à une proctectomie avec exérèse totale du mésorectum et iléostomie latérale de protection (février 2023), une radiothérapie adjuvante et une chimiothérapie adjuvante par Xeloda ; un diabète de type 2 ; une hypertension artérielle. Il suit des traitements par RAMIPRIL et METFORMINE. Il n'est pas vacciné contre le Covid-19.

Le 27 octobre 2024, il présente une toux productive. Le 29 octobre 2024, il consulte le Docteur B., médecin généraliste, en raison d'une persistance de la toux et d'une sensation de fièvre. En consultation, la tension artérielle est à 145/70 mmHg, la fréquence cardiaque à 85/min, la température à 38,7°C, la saturation en oxygène à 96 % en air ambiant. Le Docteur A. prescrit une PCR SARS-CoV-2 à Monsieur X. et un traitement symptomatique dans l'attente de l'examen.

La PCR SARS-CoV-2 réalisée le jour-même par Monsieur X. se révèle positive.

Le 2 novembre 2024, Monsieur X. se rend aux urgences de l'hôpital avec son épouse, en raison d'une dyspnée. A l'admission aux urgences, la tension artérielle est à 122/62 mmHg, la fréquence cardiaque à 74/min, la température à 37,8°C, la saturation en oxygène à 97 % en air ambiant et la fréquence respiratoire à 27/min. L'angioscanner thoracique montre un aspect de Covid-19, de sévérité modérée (10-25 %). Il n'y a pas d'argument pour une embolie pulmonaire. L'ECG s'inscrit en rythme sinusal régulier à 81/min, sans anomalie de la conduction ni de la repolarisation. Les gaz du sang montrent une hypoxémie à 64 mmHg avec un pH normal à 7,41, sans hypercapnie avec une pCO2 à 37 mmHg. L'hémogramme montre une thrombopénie à 86 G/l (130 G/l le 16/12/2021), sans autre anomalie (Hb 13,1 g/dl, PNN 2,48 G/l). La fonction rénale et le bilan d'hémostase sont normaux. La prise en charge initiale comporte une corticothérapie par DEXAMETHASONE et une anticoagulation prophylactique par ENOXAPARINE.

Monsieur X. est transféré dans le service de pneumologie pour la suite des soins. L'oxygénothérapie est à 1 l/min à l'admission. Le traitement par DEXAMETHASONE initié aux urgences est reconduit dans le service, de même que l'anticoagulation prophylactique. Une antibiothérapie probabiliste par CEFOTAXIME à dose adaptée à la fonction rénale est par ailleurs introduite le 4 novembre devant l'apparition d'une fièvre.

L'évolution est stable jusqu'à une dégradation respiratoire survenant dans la nuit du 5 au 6 novembre. L'oxygénothérapie se majore à 6 l/min le 5 novembre au soir, avec une pO2 à 76 mmHg sur les gaz du sang artériels (sous 6 l/min). Monsieur X. est transféré dans le service de réanimation médicale le 6 novembre au matin alors que le débit d'oxygénothérapie est en hausse à 10 l/min.

Une intubation orotrachéale y est réalisée le 6 novembre. La prise en charge initiale est celle d'un syndrome de détresse respiratoire aiguë sévère sur une pneumopathie SARS-CoV-2 qui nécessite la mise en place de trois décubitus ventraux. Le 10 novembre, devant la persistance de l'hypoxémie, un nouveau scanner thoracique injecté est réalisé. Il objective une atteinte parenchymateuse du SARS-CoV-2 à hauteur de plus de 75 % associée à une embolie pulmonaire lobaire inférieure droite sans signe de gravité. Une aspiration trachéale réalisée le même jour documente une surinfection bactérienne à *Pseudomonas aeruginosa*. Une antibiothérapie par TAZOCILLINE est débutée et sera relayée par la CEFTAZIDIME. Une anticoagulation curative est initiée. Devant un état d'hypoxémie réfractaire, une ECMO veino-veineuse est mise en place le 10 novembre. L'évolution se fait toutefois vers un état de choc septique sur une pneumopathie et une bactériémie à *Pseudomonas Aeruginosa* qui se complique rapidement d'un état de défaillance multiviscérale réfractaire à l'ensemble des thérapeutiques mises en place. Aucun argument échographique ne permet de suspecter une participation obstructive de l'embolie pulmonaire. Le 12 novembre, Monsieur X. décède d'un arrêt cardiocirculatoire sur asystolie non récupérée secondaire à son état de défaillance multiviscérale persistant.
