# Supplementary material for: Could ChatGPT and co. replace forensic experts? A comparative study on medical liability expertise
Source: Int J Legal Med. 2026 Mar 26;140(4):2533–41. doi: 10.1007/s00414-026-03777-2 (PMC13275606; doi:10.1007/s00414-026-03777-2)
Supplement: Supplementary file 5 — PDF 216 KB) [file 414_2026_3777_MOESM5_ESM.pdf]

Le 16 mars 2021, Madame X., 58 ans, réalise une tentative de suicide par intoxication médicamenteuse volontaire par ALPRAZOLAM et ZOPICLONE. Elle a pour seul antécédent une hypertension artérielle traitée par BIPRETERAX 10 MG/2,5 MG. Elle ne consomme pas de substance psychoactive.
Sa fille, qui l'a trouvée « endormie » en début de soirée, alerte les secours. Madame X. est transférée dans le service d'accueil des urgences du CHU le 15 mars 2021 à 20 heures 06. A son arrivée aux urgences, le score de Glasgow est à 11 (Y2V3M6). L'examen physique est normal. La surveillance clinique ne décèle pas d'altération de la vigilance au cours des heures suivantes.

Madame X. s'entretient avec le Docteur A., psychiatre, le 17 mars 2021. Le Docteur A. note :

> « *Patiente admise pour tentative de suicide par intoxication médicamenteuse volontaire.*
> *Contexte de syndrome dépressif évoluant depuis trois semaines, dans un contexte de situation professionnelle difficile. Décrit une tristesse de l'humeur, une anhédonie, des troubles des fonctions instinctuelles. Apparition progressive d'idées suicidaires ayant mené au geste d'hier, envisagé depuis plusieurs jours.*
> *A l'examen, discours cohérent, bien organisé. Ralentissement psychomoteur, faciès triste. Discours pessimiste sur sa situation professionnelle, avec persistance d'idées suicidaires toujours scénarisées par IMV mais à plus forte dose.*
> *Accepte une hospitalisation en psychiatrie. Admission en soins libres dès que possible. Traitement anxiolytique proposé dans l'immédiat.* »

Madame X. est transférée le 17 mars 2021 dans un établissement de soins psychiatriques. L'entretien d'accueil du Docteur B. conclut à un « *épisode dépressif caractérisé sévère* ». Un traitement antidépresseur par PAROXETINE est introduit, ainsi qu'un traitement anxiolytique par TRANXENE. Les suites de l'hospitalisation sont marquées par l'absence d'amélioration clinique, avec pleurs fréquents et clinophilie.

Le 23 mars 2021 à 20 heures 42, l'interne de garde est contacté par l'infirmier du service dans lequel est hospitalisée Madame X. Il note :

> « *Appel IDE pour dyspnée.*
> *Patiente qui s'est sentie essoufflée ce soir en allant en salle commune pour le repas.*
> *Pas de toux, pas de fièvre.*
> *TA 142/81 mmHg, fréquence cardiaque 125/min, température 37,2°C, SpO2 96% en air ambiant.*
> *A l'examen :*
> *Pas de tirage. Bruits du cœur réguliers, sans souffle perçu. Pas de turgescence jugulaire ni reflux hépatojugulaire.*
> *Murmure vésiculaire bilatéral et symétrique, sans bruit surajouté.*
> *Pas de signe d'anémie.*
> *Contexte de syndrome anxiodépressif. Dyspnée psychogène ? Surveillance.* »

Le 24 mars 2021, le relevé des constantes par l'infirmier est le suivant :

- A 08 heures, TA 154/85 mmHg, fréquence cardiaque 128/min, température 37,9°C, SpO2 94% en air ambiant.
- A 15 heures, TA 137/82 mmHg, fréquence cardiaque 138/min, température 38°C, SpO2 95% en air ambiant.

Le 24 mars 2021 à 18 heures 31, l'interne de garde est contacté pour une persistance de la dyspnée. La dose du traitement anxiolytique par TRANXENE est majorée. L'infirmier note : « *Patiente angoissée, se dit toujours dyspnéique. Sueurs. Appel interne de garde : augmenter le TRANXENE, rappeler si besoin.* »

Le 25 mars 2021 à 22 heures 04, un arrêt cardiorespiratoire est constaté devant témoin (la voisine de chambre de Madame X.). Un massage cardiaque externe est débuté. Le SAMU intervient. La reprise d'une activité cardiaque spontanée est obtenue. La durée de no-flow est estimée à environ 1 minute, de low-flow à 12 minutes.

Madame X. est transférée au CHU. L'angioscanner thoracique révèle une « *embolie pulmonaire proximale bilatérale, associée à des signes de cœur pulmonaire aigu* ». Une anticoagulation curative est débutée. Madame X. est hospitalisée dans le service de cardiologie.
