# Supplementary material for: Could ChatGPT and co. replace forensic experts? A comparative study on medical liability expertise
Source: Int J Legal Med. 2026 Mar 26;140(4):2533–41. doi: 10.1007/s00414-026-03777-2 (PMC13275606; doi:10.1007/s00414-026-03777-2)
Supplement: Supplementary file 6 — (PDF 205 KB) [file 414_2026_3777_MOESM6_ESM.pdf]

Madame X., 48 ans, se présente au service d'accueil des urgences du CHU le 25 avril 2024 à 13 heures 01 en raison d'une céphalée aiguë.

L'infirmière d'accueil et d'orientation relève les constantes suivantes : tension artérielle 143/92 mmHg, fréquence cardiaque 84/min, saturation en oxygène 99% en air ambiant, température 37,1°C, glycémie capillaire 1,06 g/l. L'infirmière note :

> « *Apparition aujourd'hui d'une céphalée très brutale, auto-évaluée à 8/10 sur l'échelle numérique par la patiente. Constantes correctes.* »

Monsieur A., interne, note le 25 avril 2024 à 15 heures 10 :

> « *Antécédents : fibrome utérin, dépression.*
> *Traitements : brintellix, alprazolam.*
> *Allergie : 0.*
> *Mode de vie : vit avec son époux et ses deux enfants. Enseignante.*
> *Interrogatoire :*
> *Apparition ce jour de céphalées de début brutal.*
> *Céphalées holocrâniennes, intenses. Légère phonophotophobie.*
> *Pas d'antécédent similaire, pas d'antécédent migraineux.*
> *Examen physique :*
> *Cardio : bruits du cœur réguliers sans souffle, sans signe d'insuffisance cardiaque ni de thrombose veineuse profonde, membres inférieurs inflammés*
> *Pneumo : eupnéique en air ambiant, apyrétique, murmure vésiculaire bilatéral et symétrique, sans bruit surajouté, pas de toux pas d'expectoration ni d'hémoptysie.*
> *Digestif : abdomen souple, dépressible, indolore, sans trouble du transit à priori, sans nausées ni vomissements, ni hématémèse*
> *Neuro : cohérente, consciente, GCS 15, pas de déficit sensitivomoteur aux 4 membres, pas d'anomalie à l'examen des paires crâniennes, pas de syndrome méningé.* »

Le Docteur B. note le 25 avril 2024 à 17 heures 26 :

> « *Réévaluation. Constantes correctes, pas de déficit sensitivomoteur à l'examen clinique. Probable migraine. Essai antalgiques palier 1 et AINS.* »

Le Docteur B. note le 25 avril 2024 à 17 heures 26 :

> « *Amélioration sous traitement symptomatique. RAD avec ordonnance paracetamol et ibuprofene.* »

Madame X. regagne son domicile. Le 27 avril 2024 à 19 heures 52, elle consulte de nouveau dans le service d'accueil des urgences du CHU. Le Docteur C. note :

> « *Deuxième passage aux urgences en quelques jours, est venue jeudi pour céphalée aiguë de début brutal, intense. Pas d'imagerie. Sortie sous traitement symptomatique avec légère amélioration des céphalées par la suite. Ce jour présente de nouveau une céphalée soudaine très intense holocrânienne avec photophobie. Cliniquement Glasgow 15, non déficitaire. Suspicion HSA. Scanner cérébral en urgence.* »

Le scanner cérébral, réalisé le 27 avril 2024 à 21 heures 33 et complété par un angioscanner. Le compte-rendu d'interprétation du Docteur D. se conclut par :

> « *Hémorragie sous-arachnoïdienne diffuse, Fisher 3, sur anévrisme sacciforme de l'artère communicante antérieure.* »

Le 27 avril 2024 à 22 heures 41, le Docteur C. note :

> « *HSA Fisher 3. Appel neurochirugien et radiologues interventionnels => traitement endovasculaire en urgence.* »

Madame X. est hospitalisée en unité de surveillance continue neurochirurgicale pour la suite de la prise en charge. Elle quitte finalement l'hôpital le 10 mai 2024.
