# Supplementary material for: Could ChatGPT and co. replace forensic experts? A comparative study on medical liability expertise
Source: Int J Legal Med. 2026 Mar 26;140(4):2533–41. doi: 10.1007/s00414-026-03777-2 (PMC13275606; doi:10.1007/s00414-026-03777-2)
Supplement: Supplementary file 7 — (PDF 209 KB) [file 414_2026_3777_MOESM7_ESM.pdf]

Monsieur X. est âgé de 52 ans. Il fume depuis une vingtaine d'années et est atteint d'un surpoids. Il n'a pas d'autre antécédent médico-chirurgical.

Le 5 juillet 2022, il présente des céphalées intenses, pulsatiles, d'aggravation progressive. Le 7 juillet 2022, il est conduit par un proche au cabinet de son médecin traitant. Il est adressé au service d'accueil des urgences du centre hospitalier en raison de « *céphalées qui s'aggravent depuis 4 jours + difficultés mnésiques* ». Monsieur X. est admis aux urgences à 13 heures 23 et est pris en charge à 15 heures 17. L'interne note : « *Céphalées frontales pulsatiles d'aggravation rapidement progressive depuis le 04/06, pas de photophonophobie, nausées et vomissements associés. Céphalées fluctuantes plus importantes le soir et le matin. EVA à 6-7. Douleur partiellement soulagée par le dafalgan.* » Monsieur X. est apyrétique (37,4°C à 13 heures 37 et à 37,5°C à 15 heures 04). Il n'y a pas de signe de localisation neurologique, ni de syndrome méningé. Les réflexes sont normaux. La palpation des artères temporales est normale. Il existe une sensibilité à la palpation sous-orbitaire, soulevant l'hypothèse de sinusite.

Le bilan biologique ne montre pas d'anomalie. La CRP est à 2 mg/l, sans hyperleucocytose. Le scanner cérébral ne décèle pas d'anomalie intracrânienne récente ; il n'y a pas d'argument en faveur d'une thrombophlébite cérébrale.

Monsieur X. quitte les urgences le soir-même, sous traitement antalgique (paracetamol, Acupan, Tramadol). Il est conclu : « *Le diagnostic posé est : Patient de 53 ans, tableau de céphalées atypiques évoluant depuis 4 jours, angioscanner cérébral sans anomalie. Retour à domicile ce jour sous antalgie simple devant la régression du tableau douloureux. Consultation de suivi avec son MT pour discuter, en cas de poursuite ou de récidive des céphalées dans un contexte de troubles mnésiques, de la réalisation d'une IRM encéphalique (sans urgence).* »

Le 8 juillet 2022, le tableau neurologique s'aggrave. Son médecin traitant l'adresse de nouveau aux urgences du même centre hospitalier. Le courrier d'adressage mentionne : « *aux questions posées, il répond des mots qui n'ont rien à voir, il est incapable d'ouvrir une porte, reconnaître une clef, ne sait pas à quoi servent les choses. D'après son frère, il s'est énormément aggravé depuis hier. Il semble nous comprendre mais est incapable de se souvenir de ce qu'il a mangé et de ce qu'il a fait dans la journée.* »

Monsieur X. est admis aux urgences du centre hospitalier le 8 juillet 2022 à 18 heures 09. La température à de 38,6°C à 18 heures 59. Il n'y a pas de signe de gravité hémodynamique. L'examen repère une raideur de nuque. Il n'y a pas de déficit neurologique focal. Le bilan biologique, réalisé à 21 heures 22, montre une hyperleucocytose à 13,8 G/l, à polynucléaires neutrophiles (11,98 G/l). La CRP est à 1,4 mg/l. La fonction rénale est normale. Une ponction lombaire est réalisée le 9 juillet 2022 à 01 heure 35 devant une suspicion de ménigo-encéphalite. Le liquide céphalorachidien est d'aspect clair. Un traitement par Amoxicilline + Gentamicine + Céfotaxime + Aciclovir est décidé. La première dose d'Amoxicilline est administrée le 9 juillet 2022 à 04 heures 35 et celle d'Aciclovir (900 mg/08h) à 06 heures 20 (prescription faite à 03 heures 44).

La protéinorachie est à 0,21 g/l (normale inférieure à 0,50 g/l), la glycorachie à 5,8 mmol/l (pour une glycémie capillaire à 8,2 mmol/l). Les leucocytes sont à 75/mm^3 (1% de polynucléaires neutrophiles et 99% de lymphocytes/monocytes), les hématies à 124/mm^3. L'examen direct bactériologique est négative, de même que les cultures bactériologiques. La recherche de virus est négative (entérovirus, HSV-1, HSV-2, VZV).

La CEFOTAXIME est interrompue. Monsieur X. est transféré en unité de surveillance continue le 9 juillet 2022. L'AMOXICLLINE et l'ACICLOVIR sont poursuivies jusqu'à la ponction lombaire de contrôle. Entre temps, une IRM encéphalique est réalisée le 9 juillet 2022 : elle montre des anomalies de signal au niveau des lobes temporaux, prédominant à gauche, avec atteinte de la substance blanche et de la substance grise suggérant une encéphalite nécrosante à Herpès Virus. Le diagnostic est confirmé par la deuxième ponction lombaire réalisée le 10 juillet, positive à HSV1 en PCR (protéinorachie à 0,69 g/l, glycorachie à 4,4 mmol/l, 135 éléments nucleés/mm^3 (100% de lymphocytes) et 35 hématies/mm^3).

Seul le traitement par ACICLOVIR est maintenu. Monsieur X. est hospitalisé jusqu'au 16 juillet 2022 en unité de surveillance continue. A la sortie, l'examen neurologique reste préoccupant avec une désorientation temporo-spatiale persistante, une amnésie antérograde, une agnosie et une dysphasie franche. Les troubles praxiques semblent en amélioration.

Monsieur X. est hospitalisé en maladies infectieuses du 16 juillet au 25 août 2022. L'ACICLOVIR est poursuivi pour une durée totale de 14 jours. La tolérance rénale est correcte. Il persiste des troubles neurocognitifs à l'issue de l'hospitalisation. Le 21 octobre 2022, il est reçu en consultation de maladies infectieuses. Il persiste une anosognosie, des paraphasies verbales, un manque du mot.
