# Supplementary material for: Could ChatGPT and co. replace forensic experts? A comparative study on medical liability expertise
Source: Int J Legal Med. 2026 Mar 26;140(4):2533–41. doi: 10.1007/s00414-026-03777-2 (PMC13275606; doi:10.1007/s00414-026-03777-2)
Supplement: Supplementary file 8 — (PDF 226 KB) [file 414_2026_3777_MOESM8_ESM.pdf]

Monsieur X., 21 ans, n'a pas d'antécédent médico-chirurgical ni psychiatrique. Il ne consomme pas de substance psychoactive.

Le 11 mai 2023, il est conduit par ses proches dans le service d'accueil des urgences du CHU en raison d'idées délirantes. Cliniquement, le bilan somatique est normal. Le scanner cérébral ne décèle pas d'anomalie. Le bilan biologique standard est normal et la recherche de toxiques urinaires est négative. Monsieur X. est orienté vers l'équipe de psychiatrie. Le Docteur A., psychiatre, note :

> « *Patient de 21 ans sans antécédent psychiatrique personnel ni familial.*
>
> *Anamnèse : patient conduit aux urgences par son père dans un contexte d'agitation psychomotrice, avec des gestes désordonnés et une agressivité. Dégradation progressive de son état de santé mentale sur les dernières semaines avec patient paraissant de plus en plus angoissé, verbalisant des inquiétudes d'allure délirante (ses voisins le commanderaient à distance, par exemple). Bonne intégration socio-familiale et scolaire jusqu'à ce jour (étudiant en biologie).*
>
> *En entretien, idées délirantes de persécution et de thématique hallucinatoire : a le sentiment d'être menacé par des forces diaboliques, dit qu'elles lui parlent depuis une semaine. Se croit poursuivi par des démons. Parle aussi de ses voisins qui lui voudraient du mal.*
> *Désorganisation du discours et des gestes.*
> *Retentissement anxieux majeur. Impact thymique moins net.*
>
> *CAT : hospitalisation en psychiatrie en SDT. Traitement sédatif par Tercian dans l'immédiat (pas d'allongement du QTc sur l'ECG).* »

Monsieur X. est transféré le 12 mai 2023 dans le service de psychiatrie. L'évaluation initiale conclut à un épisode psychotique aigu. Le traitement par TERCIAN est maintenu initialement. Un traitement antipsychotique par RISPERIDONE est introduit le 13 mai 2023. Les idées délirantes persistent initialement puis régressent après majoration de la RISPERIDONE à la dose de 8 milligrammes par jour. Monsieur X. quitte le service de psychiatrie de S. le 5 juin 2023.

Un suivi ambulatoire est organisé au centre médico-psychologique de Saint-Etienne. Monsieur X. y est reçu en consultation par le Docteur B. le 3 juillet 2023 et le 4 septembre 2023. Il n'est pas noté de signe de décompensation à chacun de ces deux rendez-vous. Il manque en revanche son rendez-vous de consultation du 2 décembre 2023.

Le 21 décembre 2023, Monsieur X. est de nouveau conduit par son père aux urgences du CHU. Le tableau clinique est de nouveau celui d'idées délirantes. Le père de Monsieur X. décrit un retrait social de celui-ci au cours des mois précédents. Il aurait arrêté ses études. D'après son père, le traitement par RISPERIDONE aurait bien été poursuivi. Au cours de son examen, le psychiatre note également un syndrome de désorganisation. Il conclut à une « *décompensation de schizophrénie* » et à une nouvelle indication à des soins psychiatriques à la demande d'un tiers.

Monsieur X. est hospitalisé en service de psychiatrie le jour-même. Le traitement par RISPERIDONE est arrêté, et un traitement par CLOZAPINE est introduit à dose progressive par le Docteur B. Sur la NFS pré-thérapeutique du 22 décembre 2023, les leucocytes sont à 8 G/l, dont 5,4 G/l de polynucléaires neutrophiles. La NFS fait l'objet d'une surveillance hebdomadaire pendant la période d'hospitalisation, et montre la stabilité des taux de leucocytes et de polynucléaires neutrophiles. Sur le plan psychiatrique, la symptomatologie délirante disparaît sous CLOZAPINE. Un retour à domicile est organisé le 15 janvier 2024.

Le suivi ambulatoire se poursuit auprès du Docteur B. Les consultations psychiatriques de suivi ne repèrent pas de signe de décompensation psychotique. Après 18 semaines de surveillance hebdomadaire, la NFS est surveillée à un rythme mensuel. Des bilans biologiques sont réalisées aux dates suivantes :

- 7 mai 2024 : leucocytes 6,1 G/l, polynucléaires neutrophiles 3,7 G/l
- 7 juin 2024 : leucocytes 5,8 G/l, polynucléaires neutrophiles 3,5 G/l
- 8 juillet 2024 : leucocytes 6,3 G/l, polynucléaires neutrophiles 4 G/l

Le 31 juillet 2024, Monsieur X. se rend chez son médecin généraliste, le Docteur C., en raison d'une fièvre et d'une asthénie intense. L'examen clinique du Docteur C. ne repère pas de signe de gravité, ni de point d'appel à cette fièvre. La NFS réalisée au décours de la consultation montre un taux de leucocytes à 2,1 G/l et de polynucléaires neutrophiles à 0,43 G/l. Monsieur X. est adressé aux urgences du CHU par son médecin traitant pour la prise en charge de cette agranulocytose fébrile. L'évolution y est favorable sous antibiothérapie probabiliste. Le bilan étiologique de cette agranulocytose conclut à une agranulocytose médicamenteuse secondaire à la CLOZAPINE.
