# Supplementary material for: Could ChatGPT and co. replace forensic experts? A comparative study on medical liability expertise
Source: Int J Legal Med. 2026 Mar 26;140(4):2533–41. doi: 10.1007/s00414-026-03777-2 (PMC13275606; doi:10.1007/s00414-026-03777-2)
Supplement: Supplementary file 9 — (PDF 199 KB) [file 414_2026_3777_MOESM9_ESM.pdf]

Le 10 janvier 2024 à 15 heures 30, Monsieur X., né le 10/12/1961, se présente dans le service d'accueil des urgences du centre hospitalier en raison d'une lombalgie et d'une sciatique bilatérale de trajet S1 évoluant depuis deux semaines. La lombalgie est apparue brutalement, après un effort de soulèvement. Il dit avoir du mal à bouger son pied depuis quelques jours. Le traitement par Paracetamol qu'il prenait ces derniers temps ne fait plus suffisamment effet.

A son arrivée dans le service d'accueil des urgences, la tension artérielle est de 138/87 mmHg, la fréquence cardiaque de 112/min, la température de 36,7°C, la saturation en oxygène de 94% en air ambiant. Les antécédents de Monsieur X. se limitent à une hypertension artérielle, une hypertrophie bénigne de la prostate et une chirurgie méniscale du genou gauche. Le Docteur A. note :

> « *Examen physique :*
> *Anesthésie en selle.*
> *Incapable de marcher sur les pointes des pieds, sans autre déficit moteur.*
> *Hypoesthésie dans le territoire S1 bilatéral.*
> *Abolition des réflexes achilléens, réflexes rotuliens normaux, symétriques.*
> *Pas de trouble génito-sphinctérien rapporté.*
> *Toucher rectal : tonus sphinctérien conservé.*
> *Suspicion de syndrome de la queue de cheval, demande d'IRM en urgence.* »

Une IRM médullaire est réalisée le 10 janvier 2024 à 18h00. Le Docteur B. conclut :

> « *Volumineuse hernie discale comprimant les racines S1 de manière bilatérale. Intérêt d'un avis chirurgical en urgence.* »

Le Docteur C., chirurgien du rachis, est contacté par le Docteur A. Ce dernier note à 19 heures 16 :

> « *Avis Docteur C. Indication chirurgicale en urgence. Passe voir le patient. Mettre à jeun dans l'attente du bloc. Voir avec l'anesthésiste pour consultation en urgence.* »

L'intervention chirurgicale est réalisée dans la nuit du 10 au 11 janvier 2024. Le compte-rendu opératoire du Docteur C. est intitulé :

> « *Syndrome de la queue de cheval déficitaire. Laminectomie décompressive en urgence.* »

Monsieur X. est hospitalisé dans le service de chirurgie orthopédique dans les suites de l'intervention. Le 11 janvier 2024, Madame D., interne, note :

> « *J1 post-op. Douleurs soulagées par antalgiques palier 1 + 2. Persistance d'un déficit flexion plantaire des pieds.* »

Il persiste un déficit séquellaire dans le territoire S1, de manière bilatérale.
